# The Identification of Genes Important in *Pseudomonas syringae* pv. *phaseolicola* Plant Colonisation Using *In Vitro* Screening of Transposon Libraries

**DOI:** 10.1371/journal.pone.0137355

**Published:** 2015-09-01

**Authors:** Bharani Manoharan, Helen C. Neale, John T. Hancock, Robert W. Jackson, Dawn L. Arnold

**Affiliations:** 1 Centre for Research in Bioscience, Faculty of Health and Applied Sciences, University of the West of England, Frenchay Campus, Bristol, BS16 1QY, United Kingdom; 2 School of Biological Sciences, University of Reading, Reading, RG6 6UR, United Kingdom; UMBC, UNITED STATES

## Abstract

The bacterial plant pathogen *Pseudomonas syringae* pv. *phaseolicola* (*Pph*) colonises the surface of common bean plants before moving into the interior of plant tissue, via wounds and stomata. In the intercellular spaces the pathogen proliferates in the apoplastic fluid and forms microcolonies (biofilms) around plant cells. If the pathogen can suppress the plant’s natural resistance response, it will cause halo blight disease. The process of resistance suppression is fairly well understood, but the mechanisms used by the pathogen in colonisation are less clear. We hypothesised that we could apply *in vitro* genetic screens to look for changes in motility, colony formation, and adhesion, which are proxies for infection, microcolony formation and cell adhesion. We made transposon (Tn) mutant libraries of *Pph* strains 1448A and 1302A and found 106/1920 mutants exhibited alterations in colony morphology, motility and biofilm formation. Identification of the insertion point of the Tn identified within the genome highlighted, as expected, a number of altered motility mutants bearing mutations in genes encoding various parts of the flagellum. Genes involved in nutrient biosynthesis, membrane associated proteins, and a number of conserved hypothetical protein (CHP) genes were also identified. A mutation of one CHP gene caused a positive increase in *in planta* bacterial growth. This rapid and inexpensive screening method allows the discovery of genes important for *in vitro* traits that can be correlated to roles in the plant interaction.

## Introduction

*Pseudomonas syringae* is a Gram-negative plant pathogenic bacterial species with multiple pathovars that cause a number of diseases of a wide range of plants, but can also exist out of the host plant in non-agricultural environments including rivers and snow pack [1]. *P*. *syringae* pv. *phaseolicola* (*Pph*) causes halo blight disease of the common bean *Phaseolus vulgaris* [2, 3]. *Pph* is typically associated with causing disease lesions in the leaf of bean. However, like most *P*. *syringae* strains, *Pph* usually exhibits an epiphytic lifecycle phase, existing on leaf surfaces [4, 5]. Infection involves movement of *Pph* from these zones to the inside of plant tissue, usually through wounds or natural openings such as stomata. Upon arrival into the apoplast, the change in environment stimulates the activation of genes such as the type III secretion system (T3SS), which is important for subduing the plant resistance mechanisms [6]. If *Pph* can successfully evade plant detection, and subsequent triggering of resistance, the pathogen will manipulate the plant cells to obtain nutrients allowing it to replicate. The pathogen will rapidly colonise the plant tissue, usually making polysaccharides and forming biofilms [7]. The pathogen then spreads through the apoplast to colonise uninfected tissue. Eventually, the spreading lesions will cause bacteria to remerge onto the external surfaces of the plants where they can be dispersed.

Identification of the genes involved in plant colonisation and virulence has been a long term goal of *P*. *syringae* research. Different screening methods have been used to detect genes involved in colonisation and pathogenicity of *P*. *syringae*. For example, a modified *in vivo* expression technology (IVET) approach found *in planta*-expressed promoter fusions when *P*. *syringae* pv. *tomato* (*Pto*) infected *Arabidopsis thaliana*. The study found some known genes such as T3SS effectors, but it also discovered several novel genes [8]. Another approach has been to screen for virulence genes using libraries of *P*. *syringae* transposon (Tn) mutants, and subsequently assessing mutants for alterations in virulence. For example, 2000 individual Tn*5* mutants of *Pph* were inoculated into bean pods and four of these mutants lacked the ability to cause disease or induce the hypersensitivity response (HR) [9]. Similarly, when a Tn*5* library of *Pto* was screened on tomato seedlings, nine out of 920 mutants were avirulent or exhibited very mild disease symptoms, of which five also failed to induce HR [10]. In a different study, six Tn mutants were identified in *Pph* that had lost the ability to cause disease or elicit the HR on bean. These mutants were subsequently identified as insertions into the hypersensitivity response and pathogenicity (*hrp*) genes which are responsible for the bacteria’s ability to cause disease and a HR on plants [11]. Tn mutants have also been used to identify genes involved in toxin production by the bacteria, for example, 947 *Pto* DC3000 Tn*5* mutants were screened by dip-inoculation of *A*. *thaliana* plants and 37 were found to have reduced virulence [12]. Of these *Pto* Tn mutants, six were found in previously identified phytotoxin coronatine (COR) biosynthesis genes [13]. Large numbers of Tn mutants have also been used to screen for changes in epiphytic growth on the plant surface, for example, Lindow *et al*. [5] screened 5300 Tn*5* mutants of *P*. *syringae* B728A for growth on bean leaves, 82 of which had reduced population size.

All of the above screens have been carried out by inoculating the host plant with individual Tn mutants, which is time consuming and labour intensive. A method to overcome the need for large numbers of plants has been developed more recently by screening of *P*. *syringae* pv. *maculicola* ES4326 Tn mutants on *A*. *thaliana* seedlings grown in liquid media in 96-well plates [14]. Inoculation of *A*. *thaliana* seedlings with the Tn mutants during cultivation resulted in bleaching, which was directly related to virulence of the mutants. Using this approach around 12600 mutants were screened and 40 hits in a number of interesting genes were identified as having an effect on the pathogen’s ability to cause disease, including genes involved in the T3SS, flagella-based motility and periplasmic glucan biosynthesis.

In addition to being time consuming, screening bacteria on plants is also subject to considerable variation in plant responsiveness. Moreover, most of the approaches above focus on alterations in disease symptoms, which often lead only to the identification of T3SS mutants. However, *in vitro* testing can be a useful approach to identify gene systems involved in plant colonisation. This is based on the knowledge that bacteria can behave similarly *in vitro* as they do *in vivo*, so it is possible to use certain *in vitro* phenotypic tests as proxies for *in vivo* behaviour. This type of approach is also useful for considering key processes such as pathogen entry into the leaf and spread within the apoplast. It is, of course, important to acknowledge that some of these systems are almost certainly influenced by environmental signals, but we have tested the tractability of this approach. Here we report the use of Tn screens to identify changes in *in vitro* phenotypes of *Pph* and to subsequently correlate them to changes in the plant interaction. It is therefore suggested that this would be a reliable and inexpensive method for the identification of genes involved in colonisation and virulence in *P*. *syringae* and other similar pathogens of both plants and animals.

## Materials and Methods

### Bacterial strains and growth conditions

Bacterial strains and plasmids used in this study are listed in Table 1. *Escherichia coli* strains were grown at 37°C in Luria Bertani (LB, Difco) media supplemented with 15 g/L Bacteriological No.1 agar (Oxoid, UK) and *Pseudomonas* strains were grown at 25°C on Kings medium B (KB, Difco) [15] or in LB broth. Antibiotics were used at the following concentrations (μg/ml): kanamycin (Km) 50, gentamicin (Gm) 10, and nitrofurantoin (NF) 100.

**Table 1:** Bacterial strains and plasmids.

Name	Details	Reference
***P*. *syringae* pv. *phaseolicola***		
1302A	Race 4, type strain	[16]
1448A	Race 6, type strain	[16]
***E*. *coli***		
pCR2.1 TOP10F	Competent cells	Invitrogen, UK
S17-1 λpir	Containing plasmid pSCR001	[17]
**Plasmids**		
pBBR1MCS-5	Broad host cloning vector, Gm^R^	[18]
pSCR001	Carries IS-Ω -Km/hah, Km^R^	[17]
pCR2.1 cloning vector	Km^R^ Amp^R^	Invitrogen, UK

Km^R^, Amp^R^ and Gm^R^ indicate resistance to Kanamycin, Ampicillin and Gentamycin respectively.

### Transposon library construction

Tn mutant libraries of *Pph* strains 1302A and 1448A were generated following bi-parental mating with *E*. *coli* S17-1λpir carrying IS-Ω-Km/hah (donor) [17]. Essentially 500 μl of an overnight culture of the donor strain was inoculated into 10 ml of LB broth and incubated for 3 hrs. Following incubation, 30 μl of donor was mixed with 100 μl of an overnight culture of the required *Pph* strain and the mixture transferred to the centre of an LB plate before incubation at 30°C for 48 hrs. This conjugation mixture was then diluted, plated on KB + Km + NF and incubated at 25°C for 72 hrs. Single transformation mutants were selected and inoculated on KB + Km in a 48 colony grid pattern in a 9 cm petri dish. Tn colonies were numbered, as for example, 14–3.14 (strain *Pph* 1448A-plate 3.colony 14). The insertion position of the Tns in the *Pph* genome were identified using Arbitrary Primed (AP)-PCR and DNA sequencing (Eurofins Genomics) [19, 20] (see S1 Table for primer sequences). The resulting sequences were analysed using the BLAST programme from the NCBI and insertion point determined using Artemis: Genome Browser and Annotation Tool [21] (http://www.sanger.ac.uk/resources/software/artemis/).

### Screening of transposon insertion libraries

#### Colony morphology screening

Tn libraries were replica plated onto KB agar plates. The wild type (WT) control strains were spotted onto the plate separately. Each library plate was tested in triplicate and agar plates were incubated at 25°C for three days before visual observation of the colony phenotypes. Selected individual mutants were confirmed either by streak plating on to KB agar or grown overnight in liquid culture, diluted and spread onto KB agar. Plates were incubated at 25°C for 48 hr before microscopic visualization and photography.

#### Swarming motility screening

Tn libraries were replica plated onto 0.3% LB agar. The WT control strains were spotted onto the agar plate separately. Each library plate was tested in triplicate and agar plates were incubated at 25°C for 72 hr before visual observation of the colonies’ swarming motility. Selected mutants were tested for swarming motility individually. Mutants and WT strains were spotted into the centre of a 0.3% LB agar plate. Each strain was tested in triplicate and plates were incubated and observed for up to six days at 25°C.

#### Biofilm attachment screening

Two 1302A Tn library plates were replica plated into a 96-well microtitre plate containing 200 μl LB broth + Km. The control 1302A WT was tested without Km by replacing one mutant strain per plate. Plates were incubated at 25°C for seven days without shaking. The biofilm attachment assay of the 96-well plates and individual mutants was a modified protocol based on Spiers *et al*. [22]. Essentially, after seven days the bacteria attached to the microtitre plates were stained with 1% crystal violet (CV). The density of the eluted CV was determined at OD_570_ using a microtitre plate reader (FLUOstar OPTIMA, BMG Labtech, Germany). To test individual mutants, strains were incubated in 10 ml of LB broth + Km and kept static at 25°C for seven days. After seven days, attached bacteria were stained as described above with 1 ml of 1% CV.

#### *In vitro* growth rate

Three replicates of each bacterial mutant were grown overnight in LB broth + Km and diluted to 8x10^8^ CFU/ml (OD_600_ 1.0). Cells (100 μl) were subcultured into 10 ml fresh LB broth and the optical density measured and recorded. All cultures were incubated at 25°C with shaking (10g) and their optical densities measured and recorded after 16 and 24 h. Log values of growth were calculated and plotted to compare growth of WT versus knockout mutant and ensuing complemented strains.

### Plant growth conditions and pathogenicity testing

Pathogenicity tests were carried out on bean leaves (cultivars Canadian Wonder (CW) and Tendergreen (TG) and pods (cultivar unknown) as described previously [23]. Disease was observed as water-soaking lesions around the inoculation site, whereas resistance (HR) was observed as tissue browning. For *in planta* growth rates, three replicates of each cell suspension were grown overnight in LB broth + Km and diluted to 8x10^7^ CFU/ml (OD_600_ 0.1) for infiltration and 8x10^8^ CFU/ml (OD_600_ 1.0) for spray inoculations. Cells were inoculated into 10 day old bean leaves via a syringe and needle or sprayed onto both surfaces of the leaf using a perfume atomiser until running wet and allowed to dry. Plants were incubated for 48 h (infiltration) or 120 h (spray inoculations) at 23°C, 80% humidity. A 1 cm core borer was used to harvest the inoculated area, this was homogenised in ¼ Ringers solution before being diluted and spread plated onto KB + Km. Total CFU/ml were counted and plotted to compare growth of WT versus disruption mutant and ensuing complemented strains.

### Cloning and complementation

*In vitro* complementation of selected Tn mutants was carried out following PCR amplification of the disrupted genes (see S1 Table for primer sequences) and insertion of the resulting fragment into TOPO pCR2.1 (Invitrogen) by TA cloning following the manufacturer’s instructions. The resulting construct was digested with restriction enzyme EcoR1 and the fragment ligated into broad host range vector pBBR1MCS-5 [18]. These constructs were introduced into their respective mutant strain (TnC) via electroporation, carried out as per the method of Keen *et al*. [24]. An empty vector was also transformed into the strains to use as a control (TnE).

## Results and Discussion

### *In vitro* screening of *Psuedomonas syringae* pv. *phaseolicola* transposon mutants

**Fig 1:**
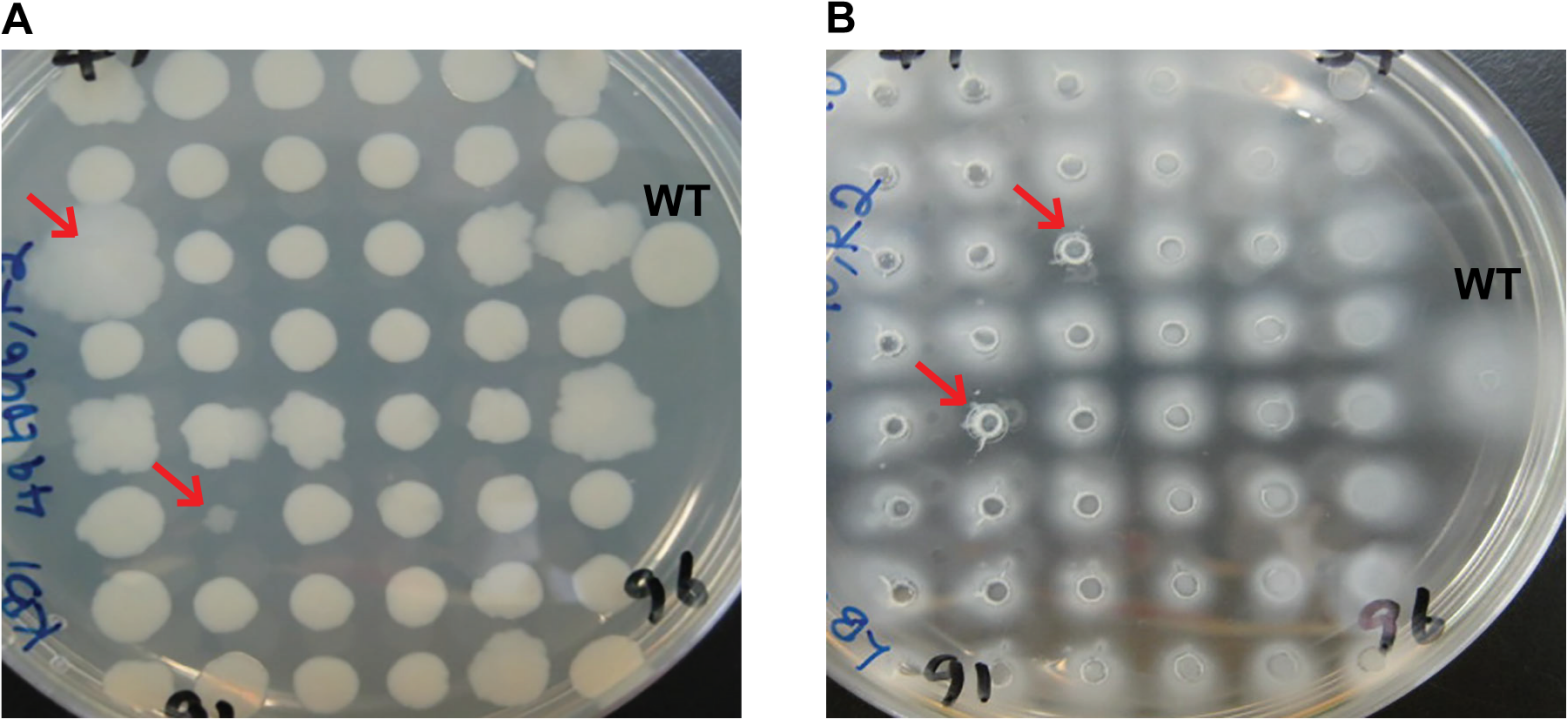
Screening of *Pseudomonas syringae* pv. *phaseolicola* 1448A transposon disruption mutants. Tn mutant colonies (48) were inoculated onto; **A.** standard agar plates and changes in colony morphology recorded after 72hr, **B.** soft agar plates and reduction in swarming ability recorded after 72hr. Selected mutants are highlighted with red arrows. WT, wild type.

**Fig 2:**
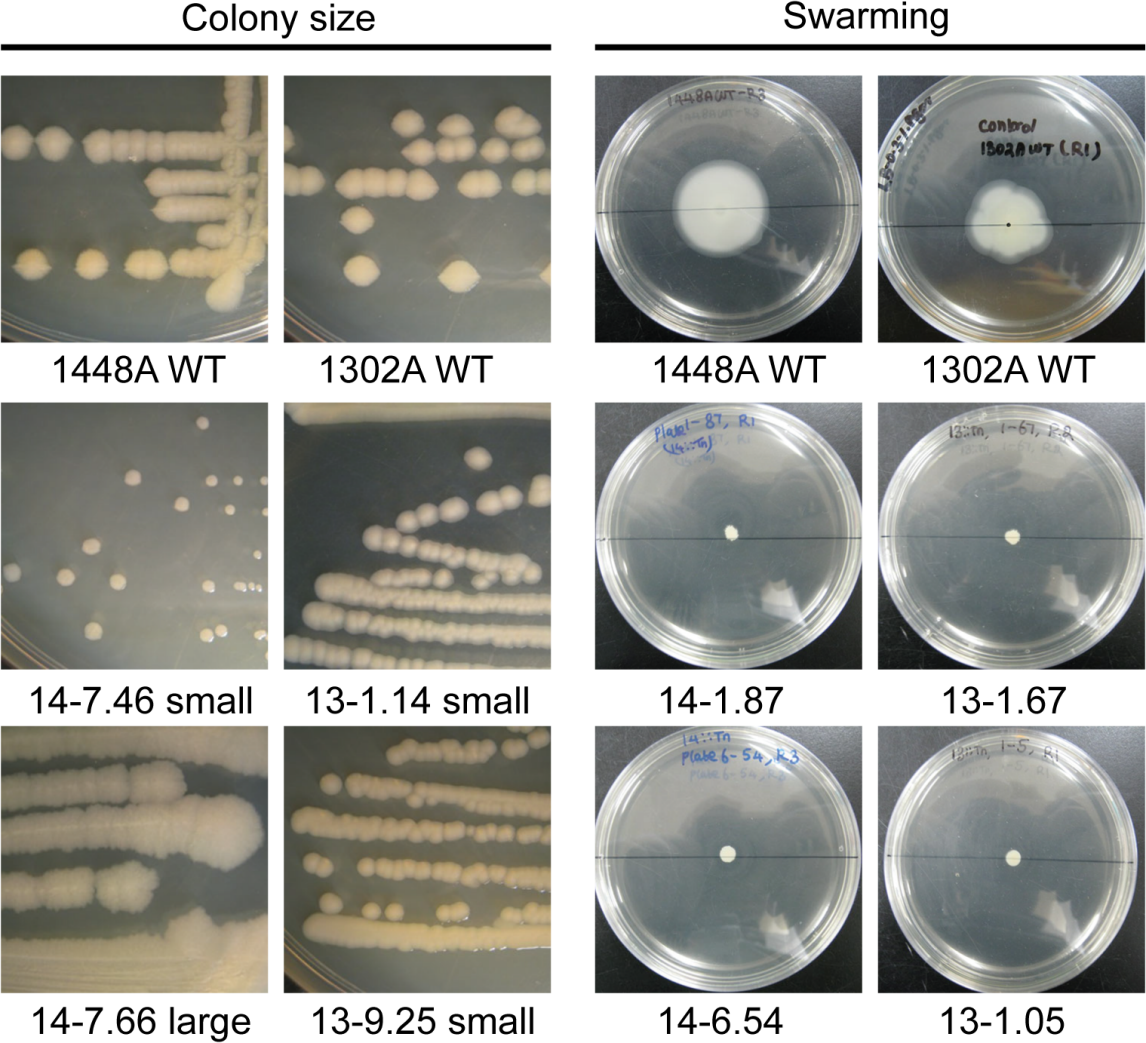
Examples of phenotypes of selected individual *Pseudomonas syringae* pv. *phaseolicola* transposon disruption mutants. Colony size selected mutants were individually streaked onto agar plates and colonies observed after 48 h incubation at the same magnification. Individual swarming mutants were inoculated into the centre of a 9 cm soft agar plate and observed after 144 h incubation. 14-, *Pph* 1448A; 13-, *Pph* 1302A; WT, wild type.

Tn mutant libraries (960 disruption mutants each) were made for *Pph* strains 1448A (causes disease on CW and TG) and 1302A (causes disease on CW and HR on TG) to screen for mutants that exhibited phenotypic changes. Firstly, colonies were screened for changes in colony morphology which may reflect changes in cell wall structure or motility that are important for colonisation of the plant. *Pph*1448A::Tn and 1302A::Tn libraries were replica plated (three replicates) onto KB plates, incubated at 25°C for 72 hr and visually compared to their equivalent WT that was also included on each 48 colony plate (Fig 1A). A total of 15 colonies for 1302A::Tn and 42 colonies for 1448A::Tn were observed as being consistently different, that is, either larger or smaller, to their WT equivalents (Table 2). The morphology of selected mutants was also confirmed by subsequently plating out the mutants individually: see Fig 2 for examples.

**Table 2:** Number of selected *Pseudomonas syringae* pv. *phaseolicola* transposon disruption mutants from 960 screened for each phenotype.

Phenotypic screening	*Pph* 1302A	*Pph* 1448A
Reduction in swarming motility	40	57
Small colonies	11	17
Large colonies	4	25
Reduced biofilm formation	6*	NT

NT—Not tested.

*950 mutants screened.

The mutant libraries as above were also screened for strains exhibiting altered ability to swarm on soft agar, to identify genes potentially involved in spreading motility, which has been shown to be important for virulence on the plant [25]. Mutant and wildtype strains were replica plated onto soft agar plates to observe swarming motility (movement over the agar surface [26]). Three replicates were carried out for each set of 48 mutant colonies and the plates were incubated at 25°C for 72 hr and visually compared to their equivalent WT (Fig 1B). Mutants that showed a difference to WT on all three replicate plates were individually tested to confirm their altered phenotype (Fig 2). This led to the selection of 97 mutants that showed a reduction in the swarming phenotype (Table 2). No increase in swarming was observed with any of the Tn mutants.

**Fig 3:**
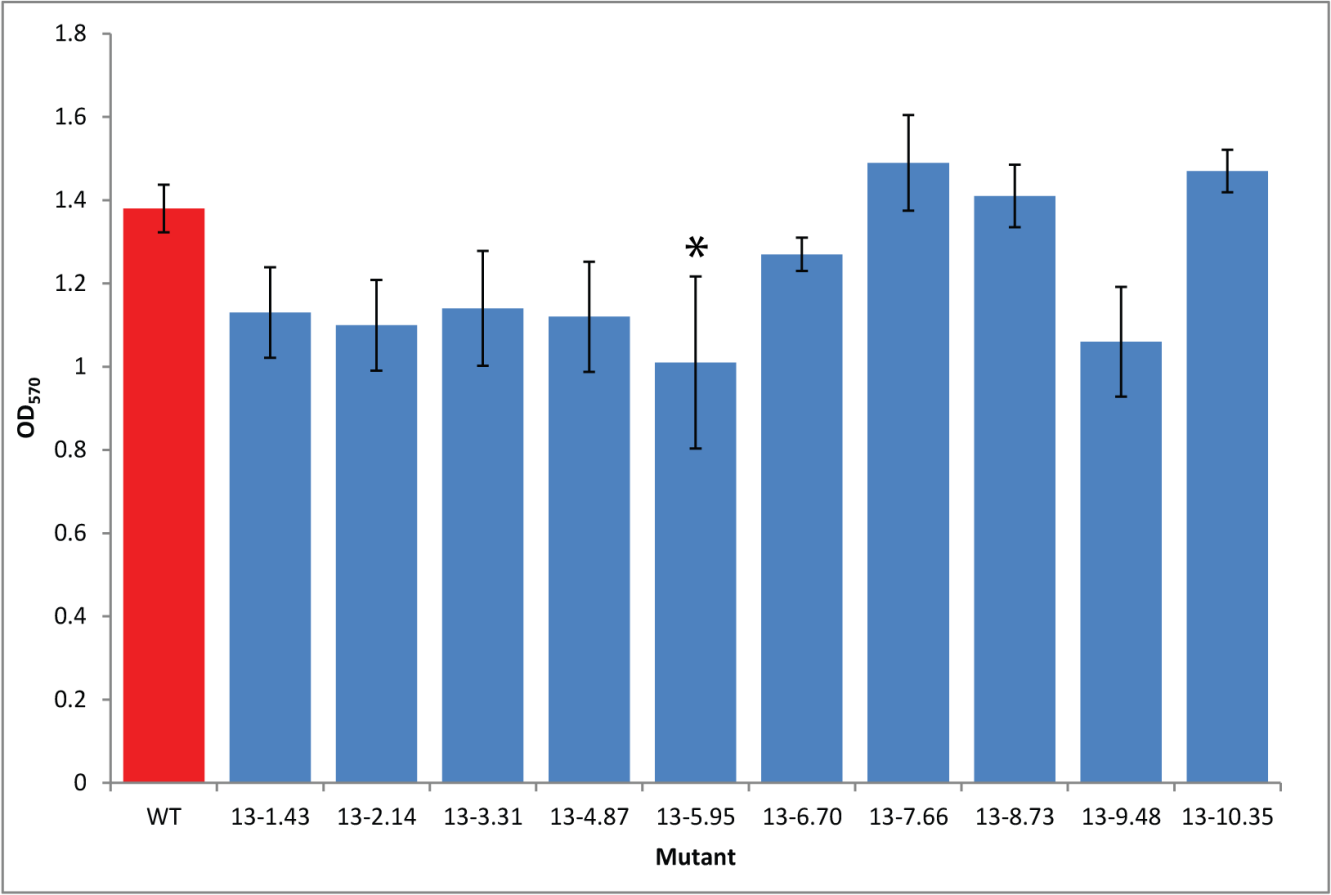
*Pseudomonas syringae* pv. *phaseolicola* transposon disruption mutants selected in a biofilm attachment assay. Transposon mutants and *Pph* 1302A wild type strain (WT) were cultured static in 10 ml broths. After seven days crystal violet was used to measure (OD_570_) the attachment of the cells to the culture vessel surface. Error bars represent standard error of the mean of three biological experimental replicates. *above bars indicate a significant difference compared to WT at p<0.05 assessed by a Student’s t-test.

A screen was also used to identify mutants exhibiting reduced adherence to surfaces, a precursor to production of biofilms. Biofilm formation by bacteria is a key strategy in the colonisation of natural environments such as plants [27]. Attachment of bacteria to surfaces is the first stage in the formation of a biofilm and this can be measured by detecting the presence of cells attached to a surface using Crystal Violet (CV) staining [22]. The 1302A::Tn library was replica plated into 96 well plates containing LB media for biofilm assay. One colony from each 96 well plate was removed and replaced with 1302A WT. For the biofilm assay, after seven days, bacteria attached to the sides of the wells were stained with CV, the concentration of which was then measured spectrophotometrically. For each 96 well plate, one mutant that had the highest attachment and one that had lowest attachment compared to WT were selected and tested individually (30 ml vial, 10 ml LB). Individual tests showed that none of the mutants were attaching more than WT which was inconsistent with the initial assay. However, of the 10 mutants selected as having lower attachment in the initial screen, six appeared to be reduced compared to WT although in only one of these was the difference statistically significant at p<0.05 (Fig 3). The six selected biofilm mutants were included in the sequence analysis of the selected mutants (below).

### Sequence analysis of selected *Pseudomonas syringae* pv. *phaseolicola* transposon mutants

**Fig 4:**
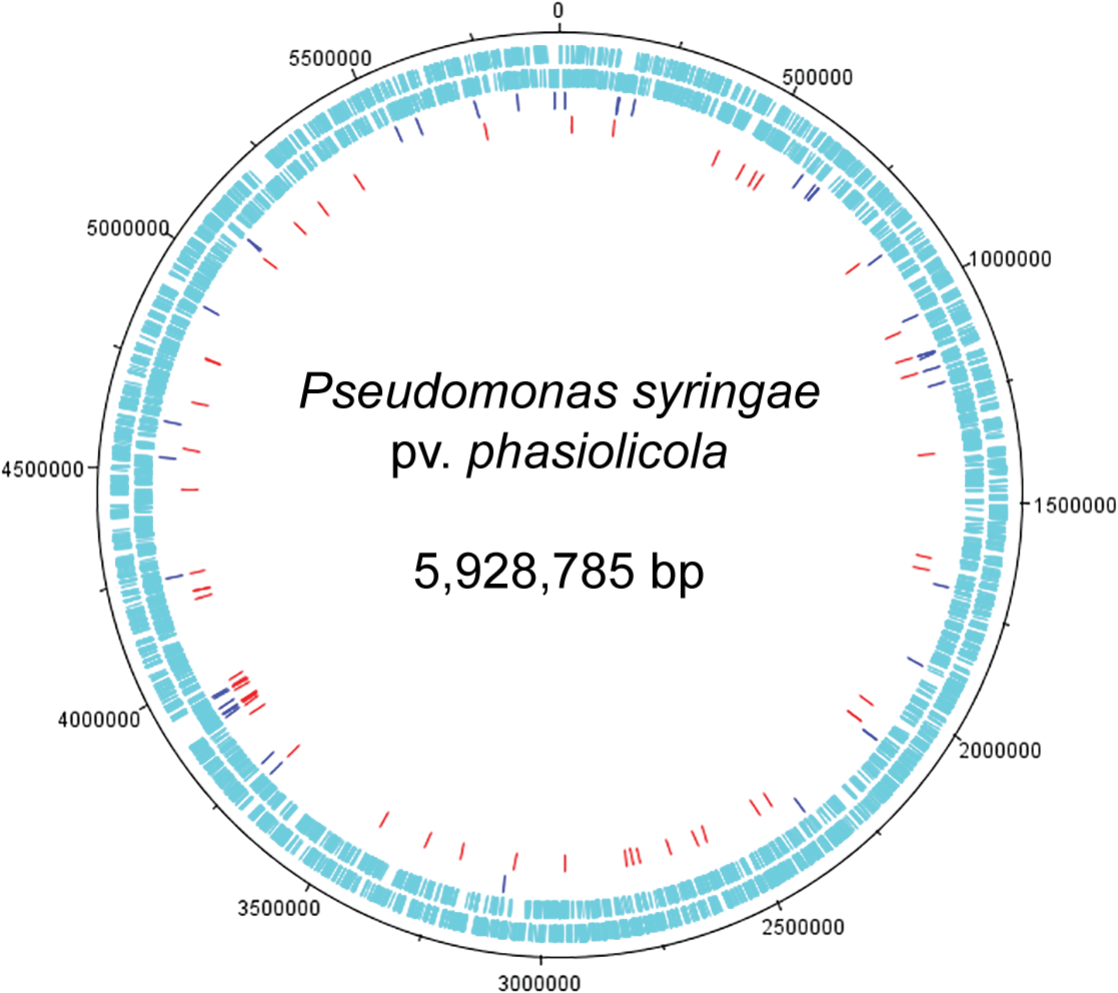
Insertion points of transposon hits on a map of the circular chromosome of *Pseudomonas syringae* pv. *phasiolicola* 1448A. Outer two circles show positions of protein-coding genes on the plus and minus strands. Red dashes, *Pph* 1448A Tn hits (56); blue dashes, *Pph* 1302A Tn hits (41). The genome map was generated by using the DNAplotter [28].

From the phenotypic screens of the Tn mutant libraries, 160 mutants were selected as showing differences to the WT strain. The 160 selected mutants consisted of 106 individual Tn mutants as some mutants were selected in multiple screens. DNA sequences were obtained from 104 of the 106 mutants (we failed on multiple occasions to obtain sequence from two mutants) and the insertion points of the Tn in the *P*. *syringae* 1448A genome were determined (S2 Table). The Tn hits were plotted on to a genome map of *Pph* 1448A using DNAPlotter (Fig 4). The map of the Tn hits shows a diverse distribution of gene knockouts around the genome, with a Tn rich area around 3,900,000 bp, which corresponds to the flagella gene cluster.

Overall most of the Tn hits were in chromosomal genes, but six Tn hits were located on plasmids and one 1302A mutant (13–10.68) had the transposon located in genomic island PPHGI-1 [29]. One of the plasmid Tn hits was to the large plasmid of 1448A and five of the plasmid hits were from the Tn library of *Pph* strain 1302A, which doesn’t currently have a genome sequence deposited. These five plasmid mutations were found on genes annotated on plasmids in *P*. *syringae* B76, *savastanoi* 3335, *maculicola* ES4326 and *Pto* DC3000.

Some genes had Tn insertions identified in the same gene from the different screening methods used here, for example, mutants (13–1.10 and 13–1.12) of 1302A were found from two different screens, for swarming and large colony formation, and the mutated gene is a mannosyltransferase. Also some genes had Tn insertions identified in the same gene from both strains tested, for example, one 1448A mutant and two 1302A mutants (14–6.14, 13–3.09, 13–9.25) had an insertional inactivation of the same OmpA domain protein, but each mutant was found via different phenotypic screens, large colony, swarming ability and small colony respectively. OmpA domain proteins are a family of outer membrane proteins found mainly in Gram-negative bacteria and have been implicated in pathogenicity, for example in bacterial adhesion [30]. However, it is interesting to also note that this OmpA domain protein, PSPPH_0123, has been identified as belonging to the type VI secretion system (T6SS), and labelled as *impL* in *Pph* 1448A [31]. The T6Ss secretion systems of Gram-negative bacteria is known to translocate effector proteins into eukaryotic host cells [31] and play a role in bacterial competition [32]. Tn hits in outer membrane proteins could be expected to affect the bacterial cell surface, which may then influence colony size and ability to swarm as was observed in our screen. It was interesting to observe, however, that the mutation in 1302A strain 13–9.25 made the colony smaller while one of the 1448A mutations in the same gene in 1448A 14–6.14 made the colony larger. We also found a number of hits in putative membrane proteins from different screens (14–6.69 and 13–1.01, large colony; 13–1.19, reduced swarming). Some screens, for example small colony size, found a number of hits in related genes. For example four hits were found in *pyr* genes (14–5.32, *pyrB*; 14–7.80, *pyrD*; 13–5.35, *pyrF*; 13–5.76, *pyrF*). A number of mutations (11) were identified in genes annotated as ‘transporters’, these came from mutants of both strains and from all the phenotypic screens described.

A number of Tn insertions were identified in genes involved in motility. Seventeen Tn insertions were found in genes involved in the flagella biosynthesis system and associated chemotaxis genes (Table 3), all of which were selected on the basis of reduced swarming ability. Flagella are appendages conferring motility for a number of bacteria [33]. Fourteen Tn mutants described in this study were part of the flagella gene regulon and included mutation of nine genes involved in the flagellum motor/switch (*fliN*, *fliM*), hook complex (*fliKI*, *flgE*, *flgD*), the flagellar export pathway (*flhB*, *fliO*) and a transcription regulator (*fleS*) [34]. Schreiber *et al*. [14] also found a number of Tn hits in flagellar genes in their *in vitro* screen of *P*. *syringae* pv. *maculicola* Tn mutants against *A*. *thaliana* seedlings. They found significant reductions in *in planta* growth compared to WT when the mutants were inoculated onto plants by spray inoculation [35] but not by pressure infiltration.

**Table 3:** *Pseudomoas syringae* pv. *phaseolicola* transposon insertions mutants identified in the flagellar and associated chemotaxis genes.

Tn mutant	Locus	Gene	Product
13–1.42	PSPPH_0554		MotA family motility protein
14–6.54	PSPPH_3360	*cheA2*	Chemotaxis sensor histidine kinase CheA
13–8.35	PSPPH_3361	*cheZ*	Chemotaxis protein CheZ
14–7.41 14–10.74 13–5.78	PSPPH_3367	*flhB*	Flagellar biosynthetic protein FlhB
13–10.60	PSPPH_3371	*fliO*	Flagellar protein FliO
14–10.63	PSPPH_3372	*fliN*	Flagellar motor switch protein FliN
14–4.52	PSPPH_3373	*fliM*	Flagellar motor switch protein FliM
14–6.51	PSPPH_3375	*fliK*	Flagellar hook-length control protein FliK
13–10.79	PSPPH_3386	*fleS*	Flagellar sensor histidine kinase FleS
14–9.76	PSPPH_3398	*flgJ*	Peptidoglycan hydrolase FlgJ
14–10.54 13–1.67	PSPPH_3405	*flgE*	Flagellar hook protein FlgE
14–2.59	PSPPH_3406	*flgD*	Basal-body rod modification protein FlgD
13–1.05 14–1.87	PSPPH_3414		Flagellar basal-body P-ring formation protein FlgA, putative

13-, *Pph* 1302A; 14-,*Pph* 1448A.

Of the three remaining motility-related mutants, two strains had Tn mutations in genes associated with chemotaxis (14–6.54, *cheA2*; 13–8.35, *cheZ*). CheA is a histidine protein kinase and CheZ is a signal terminator and both are part of the chemotaxis signalling transduction pathway that connects environmental signals to the flagella and affects the rotation and direction of movement of the bacteria [36, 37]. CheA accepts an activation signal from methyl-accepting chemotaxis proteins (MCPs) via CheW and sets off a signalling cascade that result in the stimulation of the clockwise rotation of the flagella. CheZ allows rapid termination of the signal and therefore gradient sensing. The third mutation was found in *motA*; MotA (13–1.42) is one of four cytoplasmic membrane proteins that act as motor proteins at the base of the flagellum in *P*. *syringae* pathovars. A deletion mutant of all three genes has been shown to not only completely abolish flagella-associated swarming and swimming motilities, but also reduce the ability to cause disease in tobacco leaves by *P*. *syringae* pv. *tabaci* [25]. A number of these 104 mutants are described in more detail below.

### Detecting alterations in virulence associated with the mutations

A key test of our approach was to determine whether any of the Tn mutants identified in the *in vitro* proxy phenotype screens corresponded to alterations in *in planta* growth and/or symptom defects. We therefore used bought-in bean pods for the tests and confirmed that the 1302A and 1448A WT strains caused HR and disease symptoms, respectively, in pods (S1 Fig). All 106 selected Tn mutants were tested in the bean pods. None of the 1302A mutants exhibited an altered response in causing a HR. However, some 1448A mutants showed a number of differences in symptoms to the WT (S2 Table). For example, of the four strains with mutations in *pyr* genes (14–5.32, *pyrB*; 14–7.80, *pyrD*; 13–5.35, *pyrF*; 13–5.76, *pyrF*), two affected symptoms on bean pods (these will be discussed in more detail later). Of the genes annotated as transporters, three reduced the symptoms of disease on bean pods; these were annotated as a major facilitator family transporter (14–6.12), a multidrug efflux transporter (14–10.90) and a putative spermidine/putrescine ABC transporter permease protein (14–6.19). This test confirmed alterations in disease symptoms which could be observed in Tn mutants selected through our *in vitro* screens.

### *In vitro* growth analysis of selected mutants

**Fig 5:**
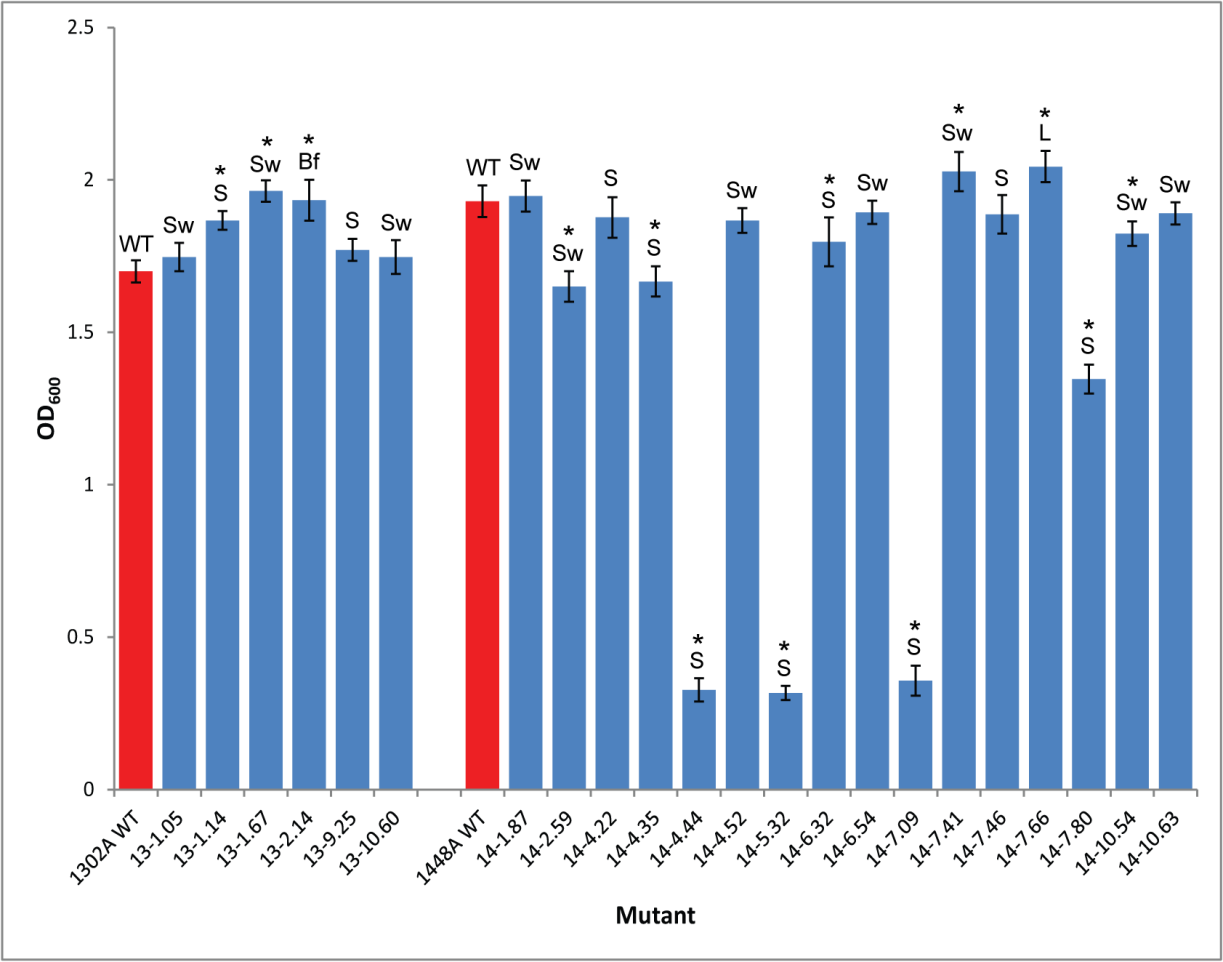
*In vitro* growth of *Pseudomonas syringae* pv. *phaseolicola* transposon mutants. Transposon mutants and wild type (WT) strains were inoculated in LB broth with shaking for 24 hr and OD_600_ recorded. Phenotypic screens: Sw, swarming reduction; S, small colony; L, large colony; Bf, biofilm formation. 14-, *Pph* 1448A; 13-, *Pph* 1302A. Error bars represent standard error of the mean of three biological experimental replicates. *above bars indicate significant differences compared to WT at p<0.05 assessed with Students t-test.

A more manageable number of Tn mutants (22) were selected for further investigation (Table 4), based on representation of each phenotypic screen, changes in their response on inoculated bean pods and some mutants with gene knockouts in known virulence factors. We first tested the *in vitro* growth of the mutants compared to the WT to determine whether the mutations fundamentally altered bacterial growth and to provide the foundation for *in planta* population growth analysis. The mutants were grown in liquid culture (LB broth) and their OD_600_ was measured after 24 hours (Fig 5). We observed that a number of the small colony mutants grew to a similar density to the WT indicating that small colony phenotype was not exclusively due to altered growth rate. However, several mutants were significantly different to the WT. Mutants 14–4.44, 14–5.32 and 14–7.09 which have gene disruptions annotated as *carA*, *carB* and *pyrB* all showed very low growth in liquid culture. Genes *carA*, *carB* and *pyrB* are involved in pyrimidine biosynthesis [38, 39]. Mutant 14–7.80 has a disruption in *pyrD* that is annotated as a dihydroorotate dehydrogenase, which is also involved in pyrimidine biosynthesis, but this does not appear to have affected *in vitro* growth as much as disruptions in the other genes.

**Table 4:** Characteristics of selected *Pseudomonas syringae* pv. *phaseolicola* transposon disruption mutants.

Mutant number	Phenotype screen	Tn insertion point (bp)^1^	Gene name/description (% for 1302A hits in 1448A genome)	Locus tag	Bean pod and leaf^2^	*In planta* growth–TG (% WT)^3^	*In planta* growth–CW (% WT)^3^
13–2.14	Biofilm		*P*.*s*. *tomato* DC3000 plasmid A, conserved hypothetical protein (88%)	PSPPH_B0022	HR	121±11	133±6
14–7.66	Large	3964031..30	Conserved hypothetical protein	PSPPH_3429	D	122±6	164±8
13–1.14	Small	13197..98	*plsC*, hdtS protein (100%)	PSPPH_0009	HR	100±2	102±2
13–9.25	Small	140944..45	*impL*, OmpA domain protein (98%)	PSPPH_0123	HR	76±5	84±10
14–4.22	Small	411911..12	*mdoG1*, periplasmic glucans biosynthesis protein MdoG	PSPPH_0359	Null	<1	<1
14–4.35	Small	4244840..39	cobyric acid synthase CobQ	PSPPH_3697	Null	<1	<1
14–4.44	Small	479168..69	*carA*, carbamoyl-phosphate synthase, small subunit	PSPPH_4203	Null	<1	<1
14–5.32	Small	537617..18	*pyrB*, aspartate carbamoyltransferase	PSPPH_0473	Null	<1	<1
14–6.32	Small	2582863..64	cobalamin synthesis protein/P47K family protein	PSPPH_2224	Null	105±7	107±2
14–7.09	Small	4791135..36	*carB*, carbamoyl-phosphate synthase, large subunit	PSPPH_4202	Null	<1	<1
14–7.46	Small	5082664..63	*purH*, bifunctional purine biosynthesis protein PurH	PSPPH_4449	Null	94±4	92±1
14–7.80	Small	2436984..83	*pyrD*, dihydroorotate dehydrogenase	PSPPH_2077	Null	<1	<1
13–1.5	Swarm	3947618..17	Flagellar basal-body P-ring formation protein FlgA, putative (99%)	PSPPH_3414	HR	80±11	88±3
13–1.67	Swarm	3940356..57	*flgE*, flagellar hook protein FlgE (80%)	PSPPH_3405	HR	73±9	85±7
13–10.60	Swarm	389967..66	*fliO*, flagellar protein FliO (96%)	PSPPH_3371	HR	128±12	189±9
14–1.87	Swarm	3947250..60	Flagellar basal-body P-ring formation protein FlgA, putative	PSPPH_3414	D	66±3	65±4
14–2.59	Swarm	3941641..42	*flgD*, basal-body rod modification protein FlgD	PSPPH_3406	D	68±1	70±6
14–4.52	Swarm	3901187..86	*fliM*, flagellar motor switch protein FliM	PSPPH_3373	D	61±2	65±2
14–6.54	Swarm	3887619..18	*cheA2*, chemotaxis sensor histidine kinase CheA	PSPPH_3360	D	69±5	65±2
14–7.41	Swarm	3897582..81	*flhB*, flagellar biosynthetic protein FlhB	PSPPH_3367	D	80±7	73±4
14–10.54	Swarm	3940841..42	*flgE*, flagellar hook protein FlgE	PSPPH_3405	D	68±1	71±5
14–10.63	Swarm	3900133..34	*fliN*, flagellar motor switch protein FliN	PSPPH_3372	D	64±8	73±1

^1^Insertion point, where given, is in *Pph* 1448A genome (accession number CP000058).

^2^Reaction on bean pods and leaves: HR, hypersensitive response; D, Disease; Null, no symptoms.

^3^ *In planta* growth in bean cultivar Tendergreen (TG) and Canadian Wonder (CW) after 2 days compared to cognate WT (1302A growth level was 4.10x10^6^ in TG and 1.80x10^7^ in CW whereas 1448A growth level was 1.40x10^7^ in TG and 2.30x10^7^ in CW cfu/ml).

± represent standard error of the mean of three biological experimental replicates. A value of 100% means that mutant shows equal growth to WT. Phenotypic screens: Swarm, swarming reduction; Small, small colony; Large, large colony; Biofilm, biofilm formation. WT, wild type; 13-, *Pph* 1302A; 14-, *Pph* 1448A.

A number of the selected 1302A mutants had a significantly increased growth rate *in vitro* compared to 1302A WT. These increased growth rate mutants included a swarming mutant (13–1.67) that was annotated as an insertion in the 1448A flagella gene, *flgE* (80% identical) and a mutant from the biofilm screen (13–2.14) that is 88% identical to a conserved hypothetical protein from a *Pto* DC3000 plasmid. A small colony mutant (13–1.14) also had an increased growth rate *in vitro*. Mutant 13–1.14 is annotated as *pls*C (100% identical) in 1448A and described as an HdtS protein. HdtS proteins have been described as *N*-acylhomoserine lactone (AHL) synthases that are involved in production of a quorum-sensing molecule [40]. However, Cullinane *et al*. [41] found that an *hdtS* mutant in *P*. *fluorescens* made normal levels of HSL and that in this bacterium the *hdtS* gene encodes a primary lysophosphatidic acid acyltransferase. The *P*. *fluorescens hdtS* mutant showed significantly impaired growth rate which we didn’t observe with 13–1.14 in liquid media, however, we did select it on a plate culture as a small mutant.

### *In planta* growth analysis of selected mutants

As we did not know the cultivar of the bean pods used for the initial pathogenicity screening, we analysed *in planta* growth rate of bacterial strains infiltrated into leaves of bean cv. TG and CW; the symptoms in the leaves were also observed. Table 4 shows the bean pod results (from initial screen), leaf symptoms and the *in planta* growth rates. We observed that 16 strains exhibited growth rates *in planta* that were reduced by more than 20% of the WT growth rate and three strains exhibited significantly increased growth; only three mutants exhibited near WT growth levels.

Considering first the reduced growth rate strains, six of the reduced growth rate mutants (all 1448A strains) exhibited less than 1% growth compared to the WT *in planta* and all six gave rise to a null reaction in bean pods. These corresponded to mutations in *carA*, *carB*, *pyrB*, *pyrD*, *cobQ* and *mdoG1*. Only the *carA*, *carB* and *pyrB* mutations corresponded to dramatic growth loss *in vitro* (OD of 0.3 cf 2.0 for WT); the *pyrD* mutation caused a modest *in vitro* decrease in growth (Fig 5). This suggests these four mutants have auxotrophic phenotypes critical for general cell growth. By contrast, the *cobQ* (14–4.35) and *mdoG1* (14–4.22) mutants exhibited small reduction and no change, respectively, in *in vitro* growth compared to the WT. This suggests these genes are much more critical for *in planta* growth and symptom development. The *cobQ* and *mdoG1* mutants were both found as small colony phenotypes. *cobQ* encodes a cobyric acid synthase which is involved in cobalamin (vitamin B_12_) biosynthesis [42]. MdoG encodes a periplasmic glucans biosynthesis protein and other work has shown that *Salmonella enterica* serovar Typhimurium mutants in *opgGH* (previously referred to as *mdoGH*) were compromised in virulence in mice compared to WT strain [43].

The remaining ten mutants (a mixture of 1448A and 1302A mutants) exhibited modest reductions in *in planta* growth compared to their cognate WT. The 1448A strains were still able to cause disease in TG and the 1302A strains still caused an HR, demonstrating these mutations did not alter the basic interaction phenotype. Nine of the strains were mutated in flagellum and chemotaxis genes, all found in the swarming screen and including *flgA* found in both strains (13–1.5, 14–1.87). The tenth mutant was a 1302A knockout of an OmpA-domain protein gene *impL* (13–9.25). As discussed earlier the flagellum mutants have previously been shown to be reduced in pathogenicity [34] and OmpA protein is an outer member protein and has been implicated in bacterial pathogenicity [30]. The reduction of growth *in planta* of the *impL* mutant may suggest a role for the T6SS system in *Pph* 1302A. The T6SS has previously been shown to have a role in virulence of *Pantoea ananatis* in onion plants [32].

Only three mutants exhibited significantly higher *in planta* growth than their respective WT strain. Of the three faster growing mutants, two were 1302A strains, mutated in a putative plasmid-borne conserved hypothetical protein (CHP) gene (found in biofilm screen; 13–2.14) and, in *fliO*, a flagellar protein (found in swarming screen; 13–10.60). The single 1448A higher growth mutant (14–7.66) was found because of its large colony phenotype and is mutated in a CHP gene. The CHP mutants both exhibited higher *in vitro* growth whereas the *fliO* mutant was not significantly difference from WT *in vitro*.

### Complementation of disruptions in a selection of mutants

**Fig 6:**
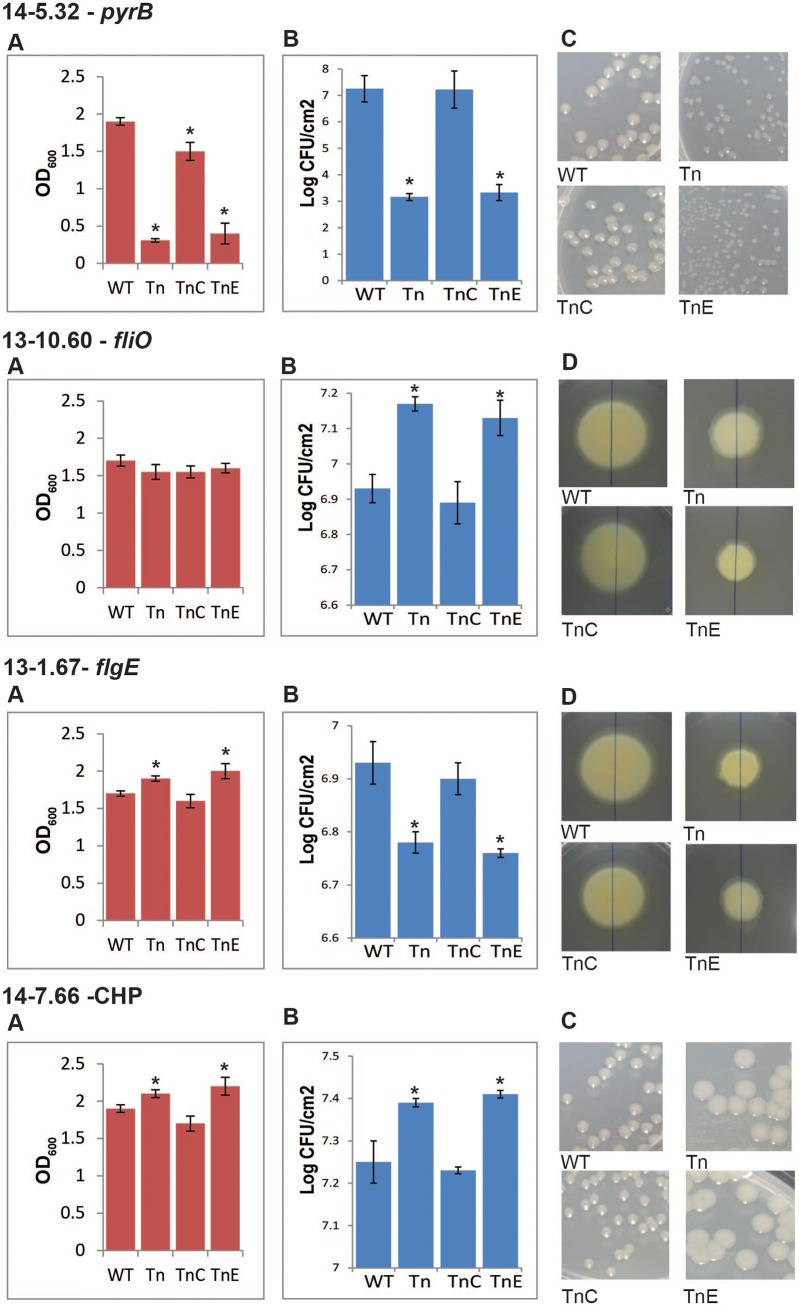
Complementation of selected *Pseudomonas syringae* pv. *phaseolicola* transposon disruption mutants. Genes identified through transposon (Tn) insertion were cloned into a broad host range vector and transformed into their respective mutant strain (TnC). An empty vector was also transformed into the strains to use as a control (TnE). A number of tests were carried out with these strains: **A.** *in vitro* growth in LB broth after 16hrs; **B.** *in planta* growth in bean cultivar Canadian Wonder after 2 days; **C.** colony size after 2 days incubation, shown at the same magnification; **D.** swarming ability in soft agar after 5 days incubation, shown at the same magnification. 14-, *Pph* 1448A; 13- *Pph* 1302A; CHP, conserved hypothetical protein. *above bars indicate significant differences compared to WT at p<0.05 assessed with Students t-test.

To validate our approach and confirm that the phenotypes observed from the mutants were due to the mutated genes, complementation experiments were undertaken. We focused on four mutants that showed significant differences to WT growth *in planta* and represented a selection of the phenotypes we had identified. For example one of these, the 1302A::*fliO* mutant, showed enhanced growth *in planta* despite still causing an HR. Each gene (open reading frame plus some flanking sequence) was amplified from the WT strain and cloned into broad host range vector pBBR1MCS-5 before electroporation into the mutant strain. An empty vector was also used as a control in all mutants tested. Complementation to WT phenotypes (or near WT) was observed for all four mutants tested, while the empty vector did not affect the mutant phenotypes, confirming that the gene mutated by the Tn was responsible for the change in phenotype (Fig 6; S3 Table).

The small colony mutant, 14–5.32, had a disrupted *pyrB* in *Pph* 1448A. This gene is annotated as an aspartate carbamoyltransferase protein involved in pyrimidine biosynthesis. The mutation in *pyrB* also leads to a very significant reduction in *in planta* growth of the bacterium. PyrB is important for bacterial survival, for example, Burns *et al*. [44] demonstrated that *pyrB* is essential for cell survival of *Helicobactor pylori* because a knockout of *pyrB* was lethal to the bacterium. A group A streptococcus (GAS) Tn library was used to identify genes important for growth and/or survival in whole human blood [36]. A *pyrB* mutant was found to be important for the fitness of GAS strain 5448 in donor blood. In addition, Tn insertion mutants of *Francisella tularensis* were screened for their inability to invade and replicate in a hepatic carcinoma cell line [45]. Eighteen mutants were identified as defective in intercellular growth in the hepatic carcinoma cell line, one of which was a mutant of *pyrB*. In this current study it is clear that the disruption in *pyrB* is severely reducing the ability of *Pph* to grow *in vitro*, so it is not surprising that it lacks the ability to grow *in planta*. However, it does confirm that mutants selected using this Tn mutagenesis approach reflect their phenotypes *in planta*.

Another mutant that was identified in the initial swarming screen was 1302A mutant 13–10.60, which has a disruption in a gene with 96% similarity to *fliO*, a flagella gene from *Pph* 1448A. Mutant 13–10.60 was selected as having a reduced ability to swarm and showed a significant increase in *in planta* growth, but no difference to WT *in vitro* growth was observed and the mutant still causes a visible HR in the resistant bean cv. TG. These results suggest that disruption of *fliO* removes some of the plants ability to restrict the growth of the bacterium. *FilO* is predicted to be part of the flagellar export pathway in *P*. *aeruginosa* [34]. If this is also true in *P*. *syringae*, then the mutation could be reducing the amount of flagellin produced in this mutant. The flg22 domain of flagellin from *P*. *syringae* pv. *tabaci*, which is encoded by *fliC*, has previously been shown to act as a microbe-associated molecular pattern (MAMP) [46]. MAMPs are conserved microbial molecules that are recognised by the plant and induce defence reactions [47]. Therefore, mutant 13–10.60 may be producing less flagellin and not triggering basal resistance in the plant, thus enabling the increased growth in early stage colonisation that we observed.

In contrast mutant 13–1.67, which was also selected initially for its reduced swarming ability, was mutated in a gene that showed 80% similarity to *flgE* of *Pph* 1448A, which is the flagellar hook protein. However this mutant (13–1.67) is reduced in its *in planta* growth rate (Fig 6) and is also slightly increased in its ability to grow *in vitro* compared to the 1448A WT. This reduced ability to grow *in planta* was observed independently in mutant 14–10.54, which also has a hit in *flgE* and also has a reduced growth rate (68% of WT) *in planta* (Table 4). Discovery of flagellar genes being involved in virulence to the plant in *Pph* is not unexpected and disruptions in flagella genes have previously been shown to reduce pathogen virulence in a number of plant pathogens [25, 33, 48, 49]. However, these studies have tended to show reduced *in planta* growth rate when the mutants were spray inoculated, rather than inoculated using an infiltration method as was carried out here. In order to compare the two inoculation methods we tested mutant 13–10.60 (*fliO*) and 13–1.67 *(flgE*) by spray inoculation method and saw no differences in the results compared to our infiltration method results (S4 Table). It may be of interest to further investigate why a mutation in the flagella hook protein is causing a significant reduction in bacterial growth once inside the leaf. Confirming the selection of these flagella mutants does however validate the method used here as a way of identifying genes responsible for flagella formation and indicates that such methodology will be useful to identify genes of interest in other less well characterised molecular systems.

Of particular interest for the discovery of new gene functions, the screens used in this work also identified disruptions in seven genes annotated as conserved hypothetical proteins (CHP) that showed altered phenotypes in colony size and swarming screens. One of these was 14–7.66 which has a disruption in a CHP in *Pph* 1448A genome and was selected as a large colony mutant in the initial screens. 14–7.66 exhibited a significant increase (110%) in *in vitro* growth and significantly increased, even higher, growth *in planta* (122% of WT). Sequence analysis of 14–7.66 showed it to be a hypothetical protein of 224 amino acids and is predicted, using InterProScan 5 [50, 51], to have a transmembrane domain and a SH3 domain. The latter would allow it to bind other proteins at proline rich regions and using String 9.1 [52], with *P*. *syringae* 1448A as a target organism, predicts possible binding partners include glycerol-3-phosphate acyltransferase and a histidine kinase, the latter suggesting that it could be involved in modulating signalling. It is possible that this CHP protein has a region external to the cell and therefore may be acting as MAMP. If this CHP can act as a MAMP it would explain why pathogenicity can increase when the protein is not functioning but further work will have to be carried out to investigate this.

## Conclusions

In this study we used random mutagenesis in combination with a series of phenotypic screens representing proxies of *in planta* growth traits to identify mutants with altered phenotypes. Our aim was to test whether the mutants showed a parallel phenotype to the plant response. Previous screening approaches have tended to screen Tn libraries of pathogenic bacteria with a plant-based assay to identify mutants of interest and then obtain DNA sequences to identify the genes involved [9, 10, 11, 12, 14]. Here we took the approach of screening for changes in phenotype that may be associated with the bacteria’s ability to interact with the plant, namely swarming ability, colony size (possibly reflecting changes in the cell wall) and biofilm formation. These assays can be done on a large scale as they are carried out with 48 mutant colonies at a time in a 9 cm Petri dishes or 96 well plates and are relatively cheap, requiring no plant growth facilities for the initial screen. With the decrease in the expense of DNA sequencing it is possible to sequence a large number of Tn hits at a reasonable cost. It was hoped that by using this approach we would find genes of interest that could be used for further investigation of the bacteria-plant interaction.

Overall we screened 1920 Tn mutants and of these 106 were selected for further analysis; of these, Tn-chromosome junction sequence was obtained from 104 strains. After further selection and characterisation we confirmed that some of these Tn hits were indeed important for the bacteria-plant interaction. For example the swarming assay produced a number of hits that were in the flagellum system and those mutants exhibited reduced *in planta* growth. Other types of genes identified included those annotated as being involved in chemotaxis, membrane proteins, nutrient biosynthesis and transporters.

Interestingly we did not find any hits in *hrp* genes or effector genes here as have been found previously when mutants have been screened directly on plants [11, 14]. However as the *hrp* system is very well characterised and not considered to be involved in our particular phenotypes used for screening, this was not unexpected. One of the more interesting results of our screen was the discovery of a CHP that was involved in restricting bacterial growth and which, when mutated, enabled the pathogen to grow to higher levels in the plant. This illustrates how this relatively simple screening technique can be used to identify previously unknown genes that may be important in the bacteria-plant interaction or potentially be targets for adaptation that increase pathogen fitness in the host. This method has generated many more genes of interest that will be useful for investigation in future studies, ultimately helping to identify new potential targets for disease control.

## Supporting Information

S1 Fig Symptoms on bean pods three days after inoculation with *Pseudomonas syringae* pv. *phaseolicola* strains. (TIF)

S1 Table Primers used in this study. (DOCX)

S2 Table Characteristics of selected *Pseudomonas syringae* pv. *phaseolicola* transposon disruption mutants. (DOCX)

S3 Table Swarming colony sizes displayed in Fig 6D. (DOCX)

S4 Table Effect of infiltration vs spray inoculation methods on *Pseudomonas syringae* pv. *phaseolicola* transposon disruption mutant growth. (DOCX)
