# Supplementary figures and images for: The Identification of Genes Important in Pseudomonas syringae pv. phaseolicola Plant Colonisation Using In Vitro Screening of Transposon Libraries
Source: PLoS One. 2015 Sep 1;10(9):e0137355. doi: 10.1371/journal.pone.0137355 (PMC4556710; doi:10.1371/journal.pone.0137355)

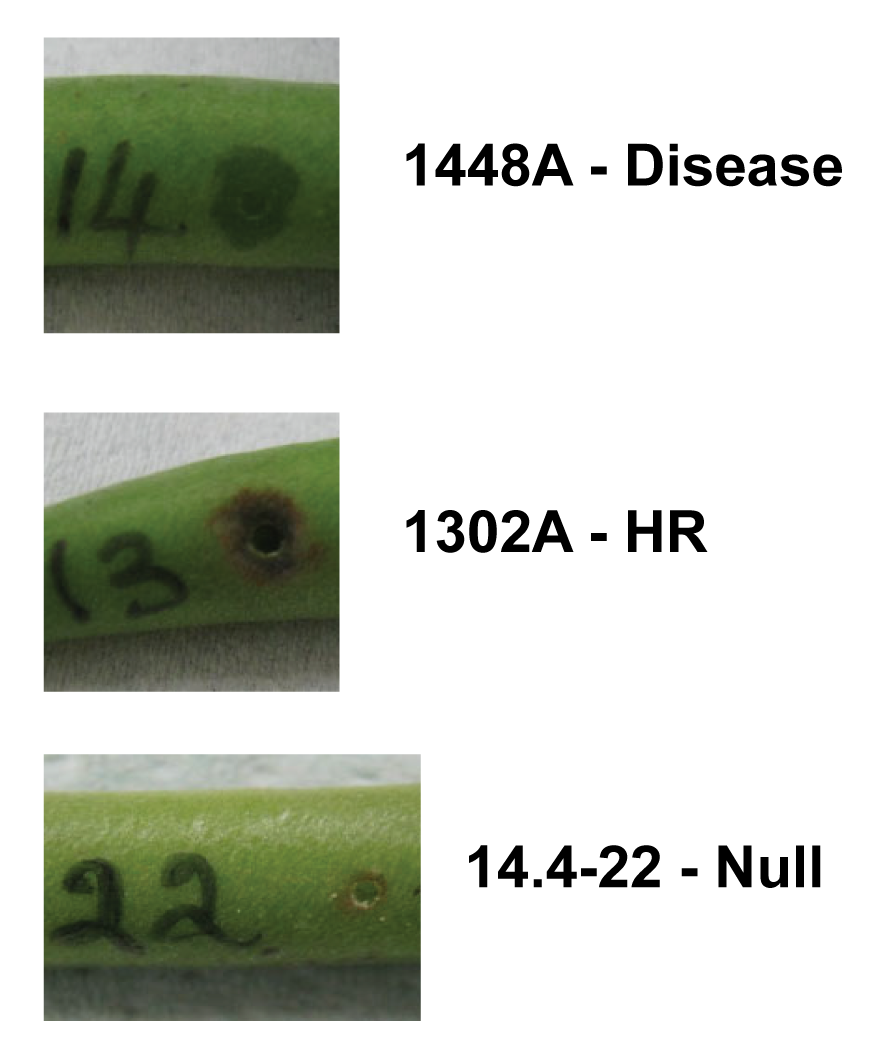

Supplement: S1 Fig — (TIF) [file pone.0137355.s001.tif]
