# Supplementary material for: The Identification of Genes Important in Pseudomonas syringae pv. phaseolicola Plant Colonisation Using In Vitro Screening of Transposon Libraries
Source: PLoS One. 2015 Sep 1;10(9):e0137355. doi: 10.1371/journal.pone.0137355 (PMC4556710; doi:10.1371/journal.pone.0137355)
Supplement: S1 Table — (DOCX) [file pone.0137355.s002.docx]

**S1 Table. Primers used in this study**

| **Primer name** | **Description** | **Sequence 5’-3’** |
| --- | --- | --- |
| Tn*pho*AII_F | Amplify the junction between the Tn IS-Ω-Km/hah and chromosome of the mutants (step one AP-PCR). | GTGTGCAGTAATATCGCCCTGAGCA |
| CEKG2A_R1 |  | GGCCACGCGTCGACTAGTACNNNNNNNNNNAGAG |
| CEKG2B_R2 |  | GGCCACGCGTCGACTAGTACNNNNNNNNNNACGCC |
| CEKG2C_R3 |  | GGCCACGCGTCGACTAGTACNNNNNNNNNNGATAT |
| Hah-1_F | Amplify the junction between the Tn IS-Ω-Km/hah and chromosome of the mutants (step two AP-PCR). | ATCCCCCTGGATGGAAAACGG |
| CEKG4_R |  | GGCCACGCGTCGACTAGTAC |
| 13-1.67F | *flgE* (PSPPH_3405) | GGCATGAATGCCGCTAATCG |
| 13-1.67R |  | CCACGGTCAACAGCGTTACG |
| 13-10.60F | *fliO* (PSPPH_3371) | GAACATGGTTCAGAAACAGCGC |
| 13-10.60R |  | CGGTTTCTCTGGACGGTCC |
| 14-5.32F | *pyrB* (PSPPH_0473) | TGCCTTTGCGGCTGTAGCC |
| 14-5.32R |  | TCTGGTGCTGATCGACGACG |
| 14-7.66F | Conserved hypothetical protein (PSPPH_3429) | CACAGTTCGCCTTACCACC |
| 14-7.66R |  | ACGGAGTCTGTTAGCTGATGG |

|  |
| --- |
