# Supplementary material for: The Identification of Genes Important in Pseudomonas syringae pv. phaseolicola Plant Colonisation Using In Vitro Screening of Transposon Libraries
Source: PLoS One. 2015 Sep 1;10(9):e0137355. doi: 10.1371/journal.pone.0137355 (PMC4556710; doi:10.1371/journal.pone.0137355)
Supplement: S2 Table — (DOCX) [file pone.0137355.s003.docx]

| **S2 Table. Characteristics of selected *Pseudomonas syringae* pv. *phaseolicola* transposon disruption mutants.** | | | | | |
| --- | --- | --- | --- | --- | --- |
| **Mutant number** | **Phenotype screen** | **Tn insertion point (bp)^1^** | **Gene name/description (1448A) (% for 1302A hits in 1448A)** | **Locus tag** | **Bean pod^2^** |
| 13-1.05 | Swarm | 3947618..17 | Flagellar basal-body P-ring formation protein FlgA, putative (99%) | PSPPH_3414 | HR |
| 13-1.10 | Swarm | 1138299..98 | Mannosyltransferase (96%) | PSPPH_0957 | HR |
| 13-1.19 | Swarm | 1143846..45 | Membrane protein, putative (90%) | PSPPH_0962 | HR |
| 13-1.38 | Swarm | 3752223..22 | Conserved hypothetical protein - conserved among plant-associated proteobacteria – down stream of hrp promoter (97%) | PSPPH_3236 | HR |
| 13-1.40 | Swarm |  | *P. syringae st*rain B76 plasmid pB76-81, hypothetical protein (91%) | (JQ418525) | HR |
| 13-1.42 | Swarm | 652603..04 | Motility protein, MotA family (100%) | PSPPH_0554 | HR |
| 13-1.57 | Swarm | 3720392..93 | *fadA*, Acetyl-CoA C-acyltransferase FadA (99%) | PSPPH_3209 | HR |
| 13-1.67 | Swarm | 3940356..57 | *flgE*, flagellar hook protein FlgE (80%) | PSPPH_3405 | HR |
| 13-3.08 | Swarm | 4903826..27 | Site-specific recombinase, phage integrase family (94%) | PSPPH_4295 | HR |
| 13-3.09 | Swarm | 143039..38 | *impL,* OmpA domain protein (96%) | PSPPH_0123 | HR |
| 13-3.33 | Swarm |  | *trbB,* *P.savastanoi* 3335 plasmid pPsv48A (93%) | PSPSV_A0061 | HR |
| 13-3.59 | Swarm |  | Partial hit with *P.s. maculicola* ES4326 plasmid pPMA4326A, hypothetical protein (94%) | PMA4326A37 | HR |
| 13-4.33 | Swarm | 5918996..97 | *parB*, chromosome partitioning protein ParB (95%) | PSPPH_5215 | HR |
| 13-5.38 | Swarm | 5095004..05 | Precorrin-3B synthase (91%) | PSPPH_4460 | HR |
| 13-5.78 | Swarm | 3897696..97 | *flhB*, flagellar biosynthetic protein FlhB (86%) | PSPPH_3367 | HR |
| 13-6.15 | Swarm | 180780..81 | Carbon-nitrogen hydrolase family protein (97%) | PSPPH_0152 | HR |
| 13-6.89 | Swarm | 4617541..42 | Downstream of - fumarate hydratase, class I, putative (99%) | PSPPH_4041 | HR |
| 13-7.35 | Swarm | 5585683..84 | Conserved hypothetical protein (95%) | PSPPH_4911 | HR |
| 13-7.45 | Swarm | 5091983..84 | CbiG protein/precorrin-3B C17-methyltransferase (99%) | PSPPH_4457 | HR |
| 13-7.89 | Swarm | 1045005..06 | *ilvC*, ketol-acid reductoisomerase (72%) | PSPPH_0874 | HR |
| 13-8.35 | Swarm | 3889543..44 | *cheZ*, chemotaxis protein CheZ (90%) | PSPPH_3361 | HR |
| 13-8.74 | Swarm | 4245891..92 | Cobalamin biosynthesis protein CobC (96%) | PSPPH_3698 | HR |
| 13-9.42 | Swarm | 878809..10 | DNA topoisomerase III (98%) | PSPPH_0752 | HR |
| 13-10.60 | Swarm | 389967..66 | *fliO*, flagellar protein FliO (96%) | PSPPH_3371 | HR |
| 13-10.68 | Swarm |  | 3-oxoacyl-acyl carrier protein synthase II, PPHGI-1, *Pph* 1302A, pph37c |  | HR |
| 13-10.73 | Swarm | 5532652..53 | *hutH2*, histidine ammonia-lyase (94%) | PSPPH_4865 | HR |
| 13-10.79 | Swarm | 3914903..04 | *fleS*, flagellar sensor histidine kinase FleS (98%) | PSPPH_3386 | HR |
| 14-1.06 | Swarm | 3862894..95 | Conserved hypothetical protein | PSPPH_3334 | D |
| 14-1.87 | Swarm | 3947259..60 | Flagellar basal-body P-ring formation protein FlgA, putative | PSPPH_3414 | D |
| 14-10.54 | Swarm | 3940841..42 | *flgE*, flagellar hook protein FlgE | PSPPH_3405 | D |
| 14-10.63 | Swarm | 3900133..34 | *fliN*, flagellar motor switch protein FliN | PSPPH_3372 | D |
| 14-10.74 | Swarm | 3897250..51 | *flhB*, flagellar biosynthetic protein FlhB | PSPPH_3367 | D |
| 14-2.29 | Swarm | 4201858..59 | UDP-glucose 4-epimerase, putative | PSPPH_3658 | D |
| 14-2.59 | Swarm | 3941641..42 | *flgD*, basal-body rod modification protein FlgD | PSPPH_3406 | D |
| 14-4.52 | Swarm | 3901187..86 | *fliM*, flagellar motor switch protein FliM | PSPPH_3373 | D |
| 14-5.24 | Swarm | 864339..40 | *pilS*, type IV pilus sensor protein PilS | PSPPH_0737 | D |
| 14-5.89 | Swarm | 4558164..65 | Acetyltransferase, GNAT family | PSPPH_3993 | D |
| 14-6.51 | Swarm | 3903176..77 | *fliK*, flagellar hook-length control protein FliK | PSPPH_3375 | D |
| 14-6.54 | Swarm | 3887619..18 | *cheA2*, chemotaxis sensor histidine kinase CheA | PSPPH_3360 | D |
| 14-7.41 | Swarm | 3897582..81 | *flhB*, flagellar biosynthetic protein FlhB | PSPPH_3367 | D |
| 14-9.10 | Swarm | 2678467..68 | Oxidoreductase, zinc-binding | PSPPH_2310 | RD |
| 14-9.76 | Swarm | 3933068..69 | *flgJ*, peptidoglycan hydrolase FlgJ | PSPPH_3398 | RD |
| 13-1.09 | Small | 663421..22 | *miaA*, tRNA delta(2)-isopentenylpyrophosphate transferase (97%) | PSPPH_0564 | HR |
| 13-1.14 | Small | 13197..98 | *plsC*, hdtS protein (100%) | PSPPH_0009 | HR |
| 13-2.03 | Small | 663423..24 | *miaA*, tRNA delta(2)-isopentenylpyrophosphate transferase (99%) | PSPPH_0564 | HR |
| 13-2.22 | Small | 1705578..77 | *srmB*, ATP-dependent RNA helicase SrmB (97%) | PSPPH_1462 | HR |
| 13-4.34 | Small | 1215819..20 | *mqo1*, malate:quinone-oxidoreductase (98%) | PSPPH_1025 | HR |
| 13-5.35 | Small | 2101648..49 | *pyrF*, orotidine 5'-phosphate decarboxylase (93%) | PSPPH_1796 | HR |
| 13-5.76 | Small | 2101620..21 | *pyrF*, orotidine 5'-phosphate decarboxylase (96%) | PSPPH_1796 | HR |
| 13-8.23 | Small | 664237..38 | *miaA*, tRNA delta(2)-isopentenylpyrophosphate (98%) | PSPPH_0564 | HR |
| 13-9.25 | Small | 140944..45 | *impL,* OmpA domain protein (98%) | PSPPH_0123 | HR |
| 13-10.23 | Small | 1898418..19 | *rluC*, ribosomal large subunit pseudouridine synthase C (93%) | PSPPH_1633 | HR |
| 13-10.88 | Small | 2340556..57 | Upstream of - *fabA*, beta-hydroxyacyl-(acyl-carrier-protein) dehydratase FabA (82%) | PSPPH_1990 | HR |
| 14-1.07 | Small | 527617..18 | Transcriptional regulator, LysR family | PSPPH_4638 | D |
| 14-1.57 | Small | 3076193..94 | Upstream of - ABC transporter, ATP-binding protein | PSPPH_2652 | D |
| 14-1.82 | Small | 2776606..07 | Conserved hypothetical protein | PSPPH_2398 | D |
| 14-2.06 | Small | 518900..01 | Class II aldolase/adducin domain protein | PSPPH_0455 | RD |
| 14-2.87 | Small | 23444..45 | *aroE*, shikimate 5-dehydrogenase | PSPPH_0027 | RD |
| 14-3.58 | Small | 4458087..88 | *glpK*, glycerol kinase | PSPPH_3899 | D |
| 14-4.22 | Small | 411911..12 | *mdoG1*, periplasmic glucans biosynthesis protein MdoG | PSPPH_0359 | Null |
| 14-4.35 | Small | 4244840..39 | Cobyric acid synthase CobQ | PSPPH_3697 | Null |
| 14-4.44 | Small | 479168..69 | *carA*, carbamoyl-phosphate synthase, small subunit | PSPPH_4203 | Null |
| 14-4.89 | Small | 4678669..70 | Sensory box histidine kinase | PSPPH_4095 | D |
| 14-5.32 | Small | 537617..18 | *pyrB*, aspartate carbamoyltransferase | PSPPH_0473 | Null |
| 14-6.32 | Small | 2582863..64 | cobalamin synthesis protein/P47K family protein | PSPPH_2224 | Null |
| 14-7.09 | Small | 4791135..36 | *carB*, carbamoyl-phosphate synthase, large subunit | PSPPH_4202 | Null |
| 14-7.46 | Small | 5082664..63 | *purH*, bifunctional purine biosynthesis protein PurH | PSPPH_4449 | Null |
| 14-7.80 | Small | 2436984..83 | *pyrD*, dihydroorotate dehydrogenase | PSPPH_2077 | Null |
| 14-8.94 | Small | 3721740..41 | *fadB*, fatty oxidation complex, alpha subunit FadB | PSPPH_3210 | D |
| 14-9.27 | Small | 1381535..36 | *gap1*, glyceraldehyde-3-phosphate dehydrogenase, type I | PSPPH_1176 | RD |
| 13-1.01 | Large | 617679..80 | Membrane protein, putative (96%) | PSPPH_0525 | HR |
| 13-1.02 | Large | 1175098..99 | Glycosyl transferase, group 1 family protein (98%) | PSPPH_0983 | HR |
| 13-1.12 | Large | 1137938..37 | Mannosyltransferase (98%) | PSPPH_0957 | HR |
| 14-2.25 | Large | 3214554..55 | *bdh1*, 3-oxoacyl-[acyl-carrier protein] reductase | PSPPH_2776 | D |
| 14-3.06 | Large | 4180741..42 | Conserved hypothetical protein | PSPPH_3635 | D |
| 14-3.37 | Large | 1134467..68 | Lipopolysaccharide ABC export system, ATP-binding protein | PSPPH_0955 | D |
| 14-6.12 | Large | 3428967..68 | Major facilitator family transporter | PSPPH_2955 | RD |
| 14-6.14 | Large | 140944..45 | *impL,* OmpA domain protein | PSPPH_0123 | D |
| 14-6.19 | Large | 2949014..15 | Spermidine/putrescine ABC transporter, permease protein, putative | PSPPH_2556 | RD |
| 14-6.55 | Large | 1641408..09 | High-affinity branched-chain amino acid ABC transporter, permease protein | PSPPH_1416 | D |
| 14-6.58 | Large | 1175138..39 | Glycosyl transferase, group 1 family protein | PSPPH_0983 | D |
| 14-6.69 | Large | 484086..87 | Membrane protein, putative | PSPPH_0419 | D |
| 14-7.28 | Large | 2039374..73 | Non-ribosomal peptide synthetase | PSPPH_1750 | D |
| 14-7.40 | Large | 3305333..34 | Penicillin-binding protein | PSPPH_2858 | D |
| 14-7.61 | Large | 1671938..39 | ABC transporter, ATP-binding/permease protein | PSPPH_1432 | D |
| 14-7.66 | Large | 3964031..30 | Conserved hypothetical protein | PSPPH_3429 | D |
| 14-7.78 | Large | 12630..31 | Large plasmid, ISPsy18, transposase, truncated | PSPPH_A0014 | RD |
| 14-8.09 | Large | 2090470..71 | Conserved hypothetical protein | PSPPH_1784 | D |
| 14-8.22 | Large | 2397739..40 | Phospho-2-dehydro-3-deoxyheptonate aldolase | PSPPH_2044 | D |
| 14-8.48 | Large | 5199623..24 | Filamentous hemagglutinin | PSPPH_4560 | D |
| 14-8.51 | Large | 2088649..50 | Acid phosphatase | PSPPH_1783 | D |
| 14-8.59 | Large | 4201462..63 | Glycosyl transferase, group 4 family protein | PSPPH_3657 | D |
| 14-8.60 | Large | 574141..42 | Conserved domain protein | PSPPH_5063 | D |
| 14-8.78 | Large | 5390751..52 | Glycine/betaine/L-proline ABC transporter, permease protein | PSPPH_4748 | D |
| 14-8.90 | Large | 2607734..33 | *malQ*, 4-alpha-glucanotransferase | PSPPH_2246 | D |
| 14-9.08 | Large | 1063126..27 | Methyl-accepting chemotaxis protein | PSPPH_0891 | D |
| 14-9.83 | Large | 2792733..34 | Lysine N6-hydroxylase/L-ornithine N5-oxygenase family protein | PSPPH_2415 | D |
| 14-10.90 | Large | 2759576..77 | Multidrug efflux transporter | PSPPH_2379 | RD |
| 13-1.43 | Biofilm | 3095872..71 | ABC transporter, periplasmic substrate-binding protein | PSPPH_2674 | HR |
| 13-2-14 | Biofilm |  | *P.s. tomato* DC3000 plasmid A, conserved hypothetical protein (88%) | PSPTO_A0040 | HR |
| 13-3.31 | Biofilm | 5829289..90 | Transcriptional regulator (96%) | PSPPH_5139 | HR |
| 13-4.87 | Biofilm | 4534590..91 | ABC transporter, ATP-binding protein (90%) | PSPPH_3973 | HR |
| 13-5.95 | Biofilm | 5730003..04 | Transcriptional regulator, AsnC family (95%) | PSPPH_5054 | HR |
| 13-9.48 | Biofilm |  | *P.s. tomato* DC3000 plasmid B, hypothetical protein (93%) | PSPTO_B0030 | HR |

^1^Insertion point, where given, is in *Pph* 1448A genome (accession number CP000058). ^2^Reaction on bean pods: HR, hypersensitive response; D, Disease; RD, reduced disease; Null, no symptoms. Phenotypic screens: Swarm, swarming reduction; Small, small colony; Large, large colony; Bio film, biofilm formation. WT, wild type; 13-, *Pph* 1302A; 14-, *Pph* 1448A.
