# Supplementary material for: The Identification of Genes Important in Pseudomonas syringae pv. phaseolicola Plant Colonisation Using In Vitro Screening of Transposon Libraries
Source: PLoS One. 2015 Sep 1;10(9):e0137355. doi: 10.1371/journal.pone.0137355 (PMC4556710; doi:10.1371/journal.pone.0137355)
Supplement: S3 Table — (DOCX) [file pone.0137355.s004.docx]

**S3 Table. Swarming colony sizes displayed in Figure 6 D.**

| **Mutant** | **WT** | **Tn** | **TnC** | **TnE** |
| --- | --- | --- | --- | --- |
| 13-10.60 | 26±2 | 18±0.5* | 25±2.5 | 16±1.5* |
| 13-1.67 | 24±0.5 | 14±1.0* | 24±0.5 | 15±0.5* |

Sizes of the two selected swarming mutants 13-10.60 and 13-1.67 after inoculation into soft agar for 5 days. Means are of three replicates ±SEM. *indicate significant differences compared to WT at p<0.05 assessed with students t-test.
