# Supplementary material for: The Identification of Genes Important in Pseudomonas syringae pv. phaseolicola Plant Colonisation Using In Vitro Screening of Transposon Libraries
Source: PLoS One. 2015 Sep 1;10(9):e0137355. doi: 10.1371/journal.pone.0137355 (PMC4556710; doi:10.1371/journal.pone.0137355)
Supplement: S4 Table — (DOCX) [file pone.0137355.s005.docx]

| **Strain** | **Infiltration**  **Log CFU/ml (48h)** | **Spray inoculation**  **Log CFU/ml (120h)** |
| --- | --- | --- |
| 1302A | 6.93±0.04 | 4.68±0.05 |
| 13-1.67 | 6.78±0.02* | 4.48±0.05* |
| 13-10.60 | 7.20±0.03* | 4.82±0.06* |

**Table S4. Effect of infiltration vs spray inoculation methods on *Pseudomonas* *syringae* pv. *phaseolicola* transposon disruption mutant growth.** Transposon mutant 13-1.67 (*flgE*) shows decreased growth and 13-10.60 (*fliO*) shows increased growth compared to wild type 1302A with both inoculation techniques. Means are of three replicates ±SEM. *indicate significant differences compared to WT at p<0.05 assessed with students t-test.
